# Non-replicative phage particles delivering CRISPR-Cas9 to target major *bla*_CTX-M_ variants

**DOI:** 10.1371/journal.pone.0303555

**Published:** 2024-05-16

**Authors:** Naiyaphat Nittayasut, Teerapong Yata, Sunisa Chirakul, Navapon Techakriengkrai, Pattrarat Chanchaithong

**Affiliations:** 1 Department of Veterinary Microbiology, Faculty of Veterinary Science, Chulalongkorn University, Bangkok, Thailand; 2 Biochemistry Unit, Department of Physiology, Faculty of Veterinary Science, Chulalongkorn University, Bangkok, Thailand; 3 Division of Bacteriology, Department of Microbiology, Faculty of Medicine, Chulalongkorn University, Bangkok, Thailand; 4 Research Unit in Food Safety and Antimicrobial Resistance, Faculty of Veterinary Science, Chulalongkorn University, Bangkok, Thailand; Nitte University, INDIA

## Abstract

Cluster regularly interspaced short palindromic repeats and CRISPR associated protein 9 (CRISPR-Cas9) is a promising tool for antimicrobial re-sensitization by inactivating antimicrobial resistance (AMR) genes of bacteria. Here, we programmed CRISPR-Cas9 with common spacers to target predominant *bla*_CTX-M_ variants in group 1 and group 9 and their promoter in an *Escherichia coli* model. The CRISPR-Cas9 was delivered by non-replicative phagemid particles from a two-step process, including insertion of spacer in CRISPR and construction of phagemid vector. Spacers targeting *bla*_CTX-M_ promoters and internal sequences of *bla*_CTX-M_ group 1 (*bla*_CTX-M-15 and -55_) and group 9 (*bla*_CTX-M-14, -27, -65, and -90_) were cloned into pCRISPR and phagemid pRC319 for spacer evaluation and phagemid particle production. Re-sensitization and plasmid clearance were mediated by the spacers targeting internal sequences of each group, resulting in 3 log_10_ to 4 log_10_ reduction of the ratio of resistant cells, but not by those targeting the promoters. The CRISPR-Cas9 delivered by modified ΦRC319 particles were capable of re-sensitizing *E*. *coli* K-12 carrying either *bla*_CTX-M_ group 1 or group 9 in a dose-dependent manner from 0.1 to 100 multiplicity of infection (MOI). In conclusion, CRISPR-Cas9 system programmed with well-designed spacers targeting multiple variants of AMR gene along with a phage-based delivery system could eliminate the widespread *bla*_CTX-M_ genes for efficacy restoration of available third-generation cephalosporins by reversal of resistance in bacteria.

## Introduction

Emergence and spread of antimicrobial resistance (AMR) are serious global health burden by retarding effectiveness of available antimicrobials used for treatment of bacterial infections [1]. Developed against acquired β-lactamase production in Enterobacterales, antimicrobial spectrum of the third-generation cephalosporins (3GCs) has been extended to tolerate the enzymes, such as *bla*_TEM-1_ and *bla*_SHV-1_ [2]. Resistance to 3GCs emerged by production of extended-spectrum β-lactamases (ESBL) that are capable of hydrolyzing 3GCs molecules, which became a major challenge due to its association with multidrug resistance from AMR gene accumulation [3]. *bla*_CTX-M_, which encodes cefotaximase-Munich (CTX-M) enzyme originated from *Kluyvera* spp., is the most abundantly distributed ESBL-encoding gene in Enterobacterales and is one of the β-lactamase family that comprises numerous variants [4]. To date, 242 variants of CTX-M have been identified and are classified into four groups based on >94% amino acid sequence identity, including CTX-M groups 1, 2, 9, and 8/25 [5, 6]. *bla*_CTX-M-15_ in group 1 and *bla*_CTX-M-14_ in group 9 are the most prevalent variants of concern worldwide, including South-East Asia [7]. In Thailand, members in groups 1 and 9, including CTX-M-9, -14, -15, -27, -55, and -65, are predominant in human patients, companion animals, swine, and poultry [8–13]. By its catalytic activity, CTX-M consequently limits clinical efficacy of penicillins, first-generation cephalosporins, and 3GCs that are antimicrobial treatment of choice for infections caused by enterobacteria [14].

Clustered regularly interspaced short palindromic repeats/CRISPR-associated proteins (CRISPR-Cas) system that acts as an adaptive immunity to prevent invasion of exogenous genetic materials in bacteria have been applied for genetic engineering biotechnology due to programmability of CRISPR and nuclease activity of Cas proteins. Among several types, CRISPR-Cas9 system that targets double-stranded DNA and subsequently causes DNA cleavage has been introduced for gene editing for reversal of resistance in bacteria to be susceptible to antimicrobial, so-called antimicrobial re-sensitization, as an alternative to antibiotic due to its ease of manipulation [15–17]. In previous studies, target DNA sequences were selected from regions in antimicrobial resistance gene to design spacer for programming CRISPR-Cas9 function and cleaving the gene that are mostly a single variant, such as *bla*_NDM-1_, *bla*_CTX-M-15_ and *bla*_TEM-1_ [18–20]. Kim et al. (2016) revealed the single target sequence of *bla*_TEM_ and *bla*_SHV_ for CRISPR-Cas9, but the consensus sequence of all *bla*_CTX-M_ variants was not detected [21]. Selection of the *bla*_CTX-M_ region as a target for re-sensitization by CRISPR-Cas9 should be an obstacle because of the high DNA polymorphisms. In addition to coding sequence, the common promoter region shared among gene variants is another target of CRISPR-Cas9 for gene inactivation in bacteria [22]. For *bla*_CTX-M_, IS*Ecp1* is preferentially associated with major variants that support gene mobilization with transposase and regulation with a strong promoter [23]. Nonetheless, inactivation of *bla*_CTX-M_ by targeting promoter region has never been evidenced to date.

Previous models of CRISPR-Cas9 construction typically targets internal sequences of antimicrobial resistance genes for re-sensitization. For a high number of variants such as *bla*_CTX-M_, the spacer is designed to target the specific variant, such as *bla*_CTX-M-15_ [24]. There is a possibility to broaden the re-sensitization spectrum to encompass more than one variant if spacer sequences are selected based on identification of the prevalent variants from epidemiological information, consensus sequences within *bla*_CTX-M_ group, and targeting the gene promoter.

**Fig 1:**
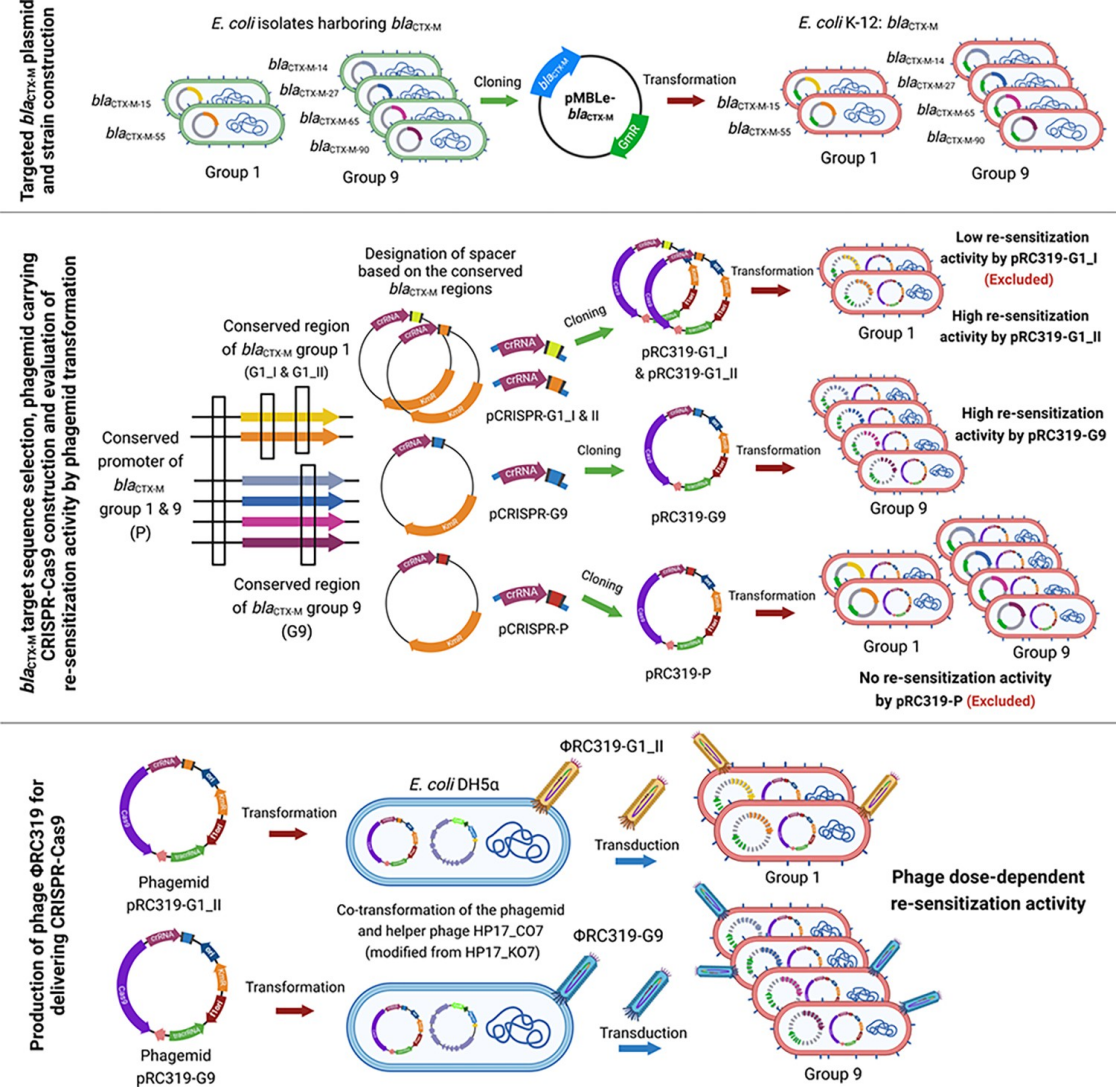
Graphical abstract.

Phagemid system, which produces non-replicative bacteriophage particles by the packaging of recombinant phagemid DNA incorporated in viral capsid facilitated by helper plasmids or helper phages. These systems consist of phagemid DNA construction and phagemid particle production. They are feasible tools for DNA delivery into bacterial cells without lytic effect [25]. This strategy has been developed and used as a prototype to deliver CRISPR-Cas9 system in a model of *E*. *coli* and *S*. *aureus* by modification of phagemid vector integrated with CRISPR-Cas9 that cause lethal effects by chromosomal cleavage and antimicrobial re-sensitization by attacking antimicrobial resistance genes [26, 27]. For a proof-of-concept of programmed CRISPR-Cas system targeting multiple variants of AMR gene, we here constructed CRISPR-Cas9 to eliminate predominant *bla*_CTX-M_ variants by using two-step cloning process into a phagemid vector for evaluation of CRISPR spacers and for production of non-replicative phage particles **(Fig 1)**.

## Materials and methods

### Bacterial strains and plasmids

*E*. *coli* DH5α (ATCC® 68233) was used for plasmid manipulation, and *E*. *coli* K-12 wildtype (ATCC® 23716) was used as recipient cells in a re-sensitization model by transformation and transduction in this study. Derivatives of both strains are described in **Table 1**, and plasmids are present in **S1 Table**. Competent cells were prepared according to the standard protocol [28].

**Table 1:** Description of bacterial strains in this study.

Strains	Description
**Clinical *E*. *coli* strains**	
*E*. *coli* CUVET16-559	*E*. *coli* strain harboring *bla*_CTX-M-15_ (GenBank accession number CP150990-CP150993)
*E*. *coli* CUVET16-465	*E*. *coli* strain harboring *bla*_CTX-M-55_ (GenBank accession number CP150994-CP150999)
*E*. *coli* CUVET16-242	*E*. *coli* strain harboring *bla*_CTX-M-14_ (GenBank accession number CP115333-CP115341)
*E*. *coli* CUVET16-394	*E*. *coli* strain harboring *bla*_CTX-M-27_ (GenBank accession number CP115330-CP115332)
*E*. *coli* CUVET18-62	*E*. *coli* strain harboring *bla*_CTX-M-65_ (GenBank accession number CP150987-CP150988)
*E*. *coli* CUVET16-819	*E*. *coli* strain harboring *bla*_CTX-M-90_ (GenBank accession number (CP150989)
***E*. *coli* DH5**α **and derivatives**	
*E*. *coli* DH5α	Competent *E*. *coli* strain for plasmid manipulation
*E*. *coli* DH5α: pRC319	Unmodified phagemid pRC319 carried by *E*. *coli* DH5α host, kanamycin resistance (Km^R^)
*E*. *coli* DH5α: pRC319-G1_I	Phagemid pRC319_G1_I: CRISPR-Cas9 carried by *E*. *coli* DH5α host, Km^R^, CRISPR RNA (crRNA): *bla*_CTX-M_ group 1
*E*. *coli* DH5α: pRC319-G1_II	Phagemid pRC319_G1_II: CRISPR-Cas9 carried by *E*. *coli* DH5α host, Km^R^, crRNA: *bla*_CTX-M_ group 1
*E*. *coli* DH5α: pRC319-G9	Phagemid pRC319_G9: CRISPR-Cas9 carried by *E*. *coli* DH5α host, Km^R^, crRNA: *bla*_CTX-M_ group 9
*E*. *coli* DH5α: pRC319-P	Phagemid pRC319_P: CRISPR-Cas9 carried by *E*. *coli* DH5α host, Km^R^, crRNA: *bla*_CTX-M_ promoter
*E*. *coli* DH5α: pRC319: pHP17_CO7	Production of ΦRC319, Km^R^ and chloramphenicol resistance (Cm^R^)
*E*. *coli* DH5α: pRC319-G1_II: pHP17_CO7	Production of ΦRC319-G1_II: CRISPR-Cas9, Km^R^ and Cm^R^, crRNA: *bla*_CTX-M_ group 1
*E*. *coli* DH5α: pRC319-G9: pHP17_CO7	Production of ΦRC319-G9: CRISPR-Cas9, Km^R^ and Cm^R^, crRNA: *bla*_CTX-M_ group 9
***E*. *coli* K-12 and derivatives**
*E*. *coli* K-12	Recipient *E*. *coli* strain for re-sensitization
*E*. *coli* K-12: pMBLe	*E*. *coli* K-12 carrying plasmid pMBLe, gentamicin resistance (Gm^R^)
*E*. *coli* K-12: *bla*_CTX-M-15_	*E*. *coli* K-12 carrying plasmid pMBLe: *bla*_CTX-M-15_, Gm^R^ and cefotaxime resistance (CTX^R^)
*E*. *coli* K-12: *bla*_CTX-M-55_	*E*. *coli* K-12 carrying plasmid pMBLe: *bla*_CTX-M-55_, Gm^R^ and CTX^R^
*E*. *coli* K-12: *bla*_CTX-M-14_	*E*. *coli* K-12 carrying plasmid pMBLe: *bla*_CTX-M-14_, Gm^R^ and CTX^R^
*E*. *coli* K-12: *bla*_CTX-M-27_	*E*. *coli* K-12 carrying plasmid pMBLe: *bla*_CTX-M-27_, Gm^R^ and CTX^R^
*E*. *coli* K-12: *bla*_CTX-M-65_	*E*. *coli* K-12 carrying plasmid pMBLe: *bla*_CTX-M-65_, Gm^R^ and CTX^R^
*E*. *coli* K-12: *bla*_CTX-M-90_	*E*. *coli* K-12 carrying plasmid pMBLe: *bla*_CTX-M-90_, Gm^R^ and CTX^R^
*E*. *coli* K-12: *bla*_CTX-M-15_: pRC319-G1_I	Co-transformant of *E*. *coli* K-12 carrying pMBLe: *bla*_CTX-M-15_ and pRC319-G1_I: CRISPR-Cas9, crRNA: *bla*_CTX-M_ group 1
*E*. *coli* K-12: *bla*_CTX-M-15_: pRC319-G1_II	Co-transformant of *E*. *coli* K-12 carrying pMBLe: *bla*_CTX-M-15_ and pRC319-G1_II: CRISPR-Cas9, crRNA: *bla*_CTX-M_ group 1
*E*. *coli* K-12: *bla*_CTX-M-55_: pRC319-G1_I	Co-transformant of *E*. *coli* K-12 carrying pMBLe: *bla*_CTX-M-55_ and pRC319-G1_I: CRISPR-Cas9, crRNA: *bla*_CTX-M_ group 1
*E*. *coli* K-12: *bla*_CTX-M-55_: pRC319-G1_II	Co-transformant of *E*. *coli* K-12 carrying pMBLe: *bla*_CTX-M-55_ and pRC319-G1_II: CRISPR-Cas9, crRNA: *bla*_CTX-M_ group 1
*E*. *coli* K-12: *bla*_CTX-M-14_: pRC319-G9	Co-transformant of *E*. *coli* K-12 carrying pMBLe: *bla*_CTX-M-14_ and pRC319-G9: CRISPR-Cas9, crRNA: *bla*_CTX-M_ group 9
*E*. *coli* K-12: *bla*_CTX-M-27_: pRC319-G9	Co-transformant of *E*. *coli* K-12 carrying pMBLe: *bla*_CTX-M-27_ and pRC319-G9: CRISPR-Cas9, crRNA: *bla*_CTX-M_ group 9
*E*. *coli* K-12: *bla*_CTX-M-65_: pRC319-G9	Co-transformant of *E*. *coli* K-12 carrying pMBLe: *bla*_CTX-M-65_ and pRC319-G9: CRISPR-Cas9, crRNA: *bla*_CTX-M_ group 9
*E*. *coli* K-12: *bla*_CTX-M-90_: pRC319-G9	Co-transformant of *E*. *coli* K-12 carrying pMBLe: *bla*_CTX-M-90_ and pRC319-G9: CRISPR-Cas9, crRNA: *bla*_CTX-M_ group 9
*E*. *coli* K-12: *bla*_CTX-M-15_: pRC319-P	Co-transformant of *E*. *coli* K-12 carrying pMBLe: *bla*_CTX-M-15_ and pRC319-P: CRISPR-Cas9, crRNA: *bla*_CTX-M_ promoter
*E*. *coli* K-12: *bla*_CTX-M-55_: pRC319-P	Co-transformant of *E*. *coli* K-12 carrying pMBLe: *bla*_CTX-M-55_ and pRC319-P: CRISPR-Cas9, crRNA: *bla*_CTX-M_ promoter
*E*. *coli* K-12: *bla*_CTX-M-14_: pRC319-P	Co-transformant of *E*. *coli* K-12 carrying pMBLe: *bla*_CTX-M-14_ and pRC319-P: CRISPR-Cas9, crRNA: *bla*_CTX-M_ promoter
*E*. *coli* K-12: *bla*_CTX-M-27_: pRC319-P	Co-transformant of *E*. *coli* K-12 carrying pMBLe: *bla*_CTX-M-27_ and pRC319-P: CRISPR-Cas9, crRNA: *bla*_CTX-M_ promoter
*E*. *coli* K-12: *bla*_CTX-M-65_: pRC319-P	Co-transformant of *E*. *coli* K-12 carrying pMBLe: *bla*_CTX-M-65_ and pRC319-P: CRISPR-Cas9, crRNA: *bla*_CTX-M_ promoter
*E*. *coli* K-12: *bla*_CTX-M-90_: pRC319-P	Co-transformant of *E*. *coli* K-12 carrying pMBLe: *bla*_CTX-M-90_ and pRC319-P: CRISPR-Cas9, crRNA: *bla*_CTX-M_ promoter
*E*. *coli* K-12: *bla*_CTX-M-15_: ΦRC319-G1_II	Transductant of *E*. *coli* K-12 carrying pMBLe: *bla*_CTX-M-15_ with ΦRC319-G1_II: CRISPR-Cas9, crRNA: *bla*_CTX-M_ group1
*E*. *coli* K-12: *bla*_CTX-M-55_: ΦRC319-G1_II	Transductant of *E*. *coli* K-12 carrying pMBLe: *bla*_CTX-M-55_ with ΦRC319-G1_II: CRISPR-Cas9, crRNA: *bla*_CTX-M_ group1
*E*. *coli* K-12: *bla*_CTX-M-14_: ΦRC319-G9	Transductant of *E*. *coli* K-12 carrying pMBLe: *bla*_CTX-M-14_ with ΦRC319-G9: CRISPR-Cas9, crRNA: *bla*_CTX-M_ group9
*E*. *coli* K-12: *bla*_CTX-M-27_: ΦRC319-G9	Transductant of *E*. *coli* K-12 carrying pMBLe: *bla*_CTX-M-27_ with ΦRC319-G9: CRISPR-Cas9, crRNA: *bla*_CTX-M_ group9
*E*. *coli* K-12: *bla*_CTX-M-65_: ΦRC319-G9	Transductant of *E*. *coli* K-12 carrying pMBLe: *bla*_CTX-M-65_ with ΦRC319-G9: CRISPR-Cas9, crRNA: *bla*_CTX-M_ group9
*E*. *coli* K-12: *bla*_CTX-M-90_: ΦRC319-G9	Transductant of *E*. *coli* K-12 carrying pMBLe: *bla*_CTX-M-90_ with ΦRC319-G9: CRISPR-Cas9, crRNA: *bla*_CTX-M_ group9

### Targeted *bla*_CTX-M_ plasmid and strain construction

*bla*_CTX-M_ variants, including *bla*_CTX-M_ group 1 (*bla*_CTX-M-15 and -55_) and *bla*_CTX-M_ group 9 (*bla*_CTX-M-14, -27, -65, and -90_), were amplified from clinical *E*. *coli* strains (**Table 1**) with ISEcp1_Prom_F, ISEcp1_Prom_R, and ISEcp1_Prom_14_R primers (**S2 Table**) and cloned into pT&A cloning vector by using T&A^TM^ cloning vector kit (Yeastern Biotech, Taiwan) before transformation into the competent *E*. *coli* DH5α by heat shock. Transformants were selected on X-gal/IPTG Luria-Bertani (LB) plates (HiMedia®, Mumbai, India) containing ampicillin (100 μg/mL, abbreviated to Am100) and confirmed by colony PCR. To construct *bla*_CTX-M_ expression system, the *bla*_CTX-M_ genes were subcloned into pMBLe vector [29], which was kindly provided from Professor Herbert Schweizer, by *Eco*RI and *Hind*III restriction endonucleases and subsequently transformed into *E*. *coli* K-12 to be target strains (*E*. *coli* K-12: *bla*_CTX-M_ strains) in re-sensitization model (**Table 1**). The transformants were selected using gentamicin (15 μg/mL, abbreviated to Gm15) and cefotaxime (2 μg/mL, abbreviated to CTX2) in LB agar and confirmed by colony PCR.

### *bla*_CTX-M_ target sequence selection and CRISPR construction

Sequences of *bla*_CTX-M-15 and -55_ (group 1), *bla*_CTX-M-14, -27, -65, and -90_ (group 9) as well as their promoters were initially aligned to observed consensus regions and protospacer adjacent motif (PAM) sequence (NGG) for Cas9 endonuclease by CHOPCHOP web server (http://chopchop.cbu.uib.no) [30, 31]. Common spacers of each *bla*_CTX-M_ group and the promoter were selected from both strands that included 20 nucleotides upstream of PAM sequences and had 25–75% GC content without nucleotide polymorphism to be crRNA. In addition, the sequences must not contain off-target sequences on *E*. *coli* K-12 genome [32].

The spacers with leading sequences [5′-AAAC(N_30_)G-3′ and 3′-CAAAA(N_30_)-5′] (**S3 Table**) were synthesized and ligated to pCRISPR vector by Golden gate cloning technique [33, 34]. Pairs of oligos were phosphorylated using T4 polynucleotide kinase (New England Biolabs, MA) and annealed before insertion into *Bsa*I-digested pCRISPR backbone in a 20-μL T4 ligase reaction. The spacer-containing pCRISPR plasmids were transformed into *E*. *coli* DH5α that were selectively grown on LB agar containing kanamycin (35 μg/mL, abbreviated to Km35) and detected by PCR and DNA sequencing of 341-bp specific fragment using primers in **S2 Table**.

### Recombinant phagemid containing CRISPR-Cas9

**Fig 2:**
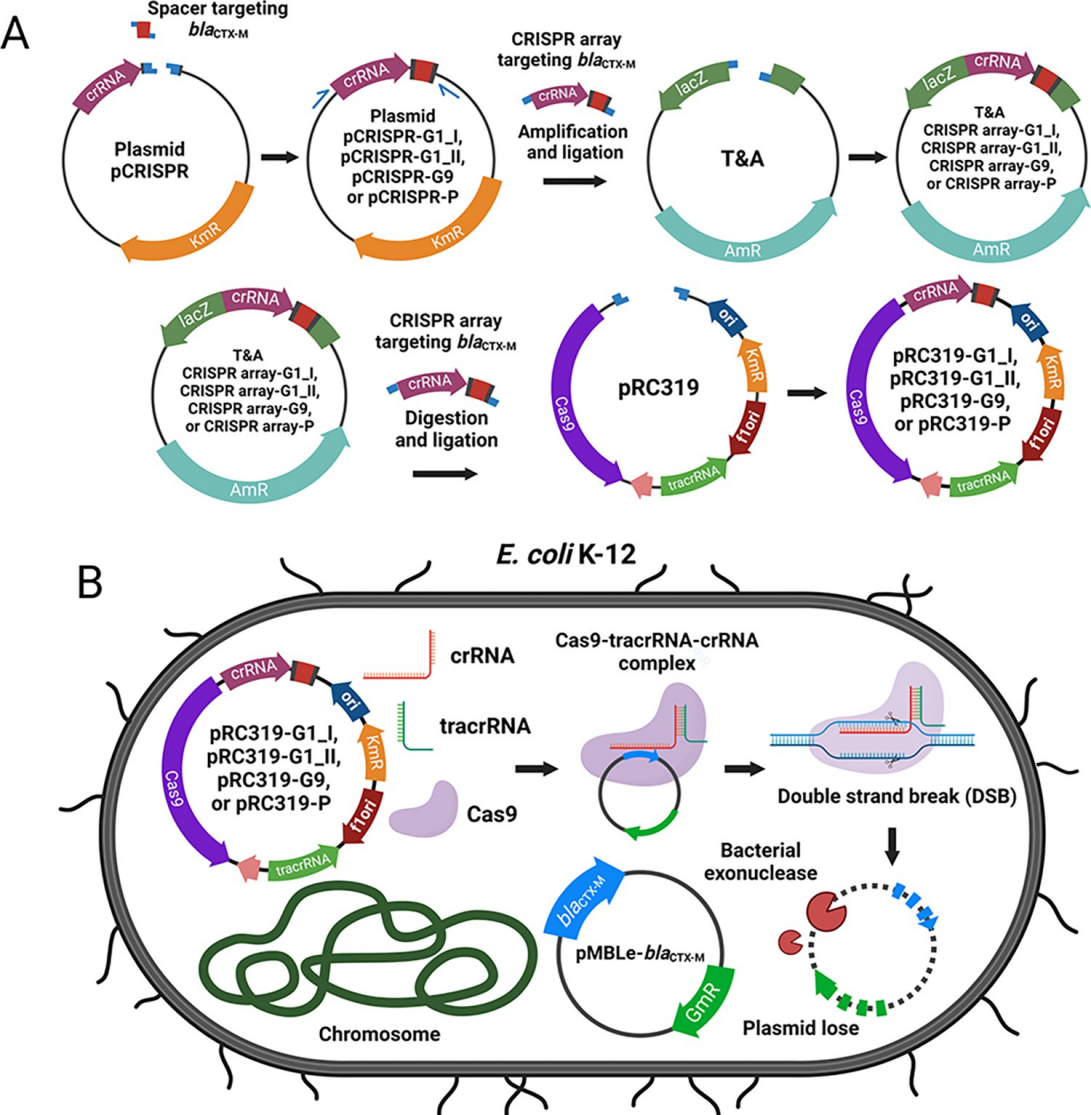
Schematic presenting workflow of plasmid construction and antimicrobial re-sensitization by transformation. (**A**) The candidate target sequence was inserted into CRISPR array on pCRISPR, and then CRISPR array was constructed into phagemid pRC319 that contained Cas9 and tracrRNA and the programmed CRISPR-Cas9 targeting *bla*_CTX-M_ genes and the promoter (**B**) The modified pRC319 containing the CRISPR-Cas9 system was transformed into *E*. *coli* K-12 carrying *bla*_CTX-M_ on pMBLe plasmid. The programmed CRISPR-Cas9 expressed functional Cas9-tracrRNA-crRNA complex to cleave pMBLe-*bla*_CTX-M_ plasmid at the target site that resulted in antimicrobial re-sensitization and plasmid clearance. The figure was generated by BioRender web software (www.app.biorender.com).

The 421-bp fragments of the inserted spacer in CRISPR region were amplified from pCRISPR-G1_I, pCRISPR-G1_II, pCRISPR-G9, and pCRISPR-P using Spc_PspXI_F and Spc_XmaI_R primers to generate the recognition sites for *Psp*XI and *Xma*I before cloning in pT&A vector and transformation into *E*. *coli* DH5α. Each CRISPR fragment from the modified pCRISPR was ligated to *Psp*XI- and *Xma*I-digested phagemid pRC319, which encodes Cas9 endonuclease and trans-activating CRISPR (tracr) RNA, by T4 ligase (**Fig 2A**) [27]. Successful transformation of pRC319-G1_I, pRC319-G1_II, pRC319-G9, and pRC319-P into *E*. *coli* were examined by growing on Km35-containing LB agar and nucleotide sequencing.

**Fig 3:**
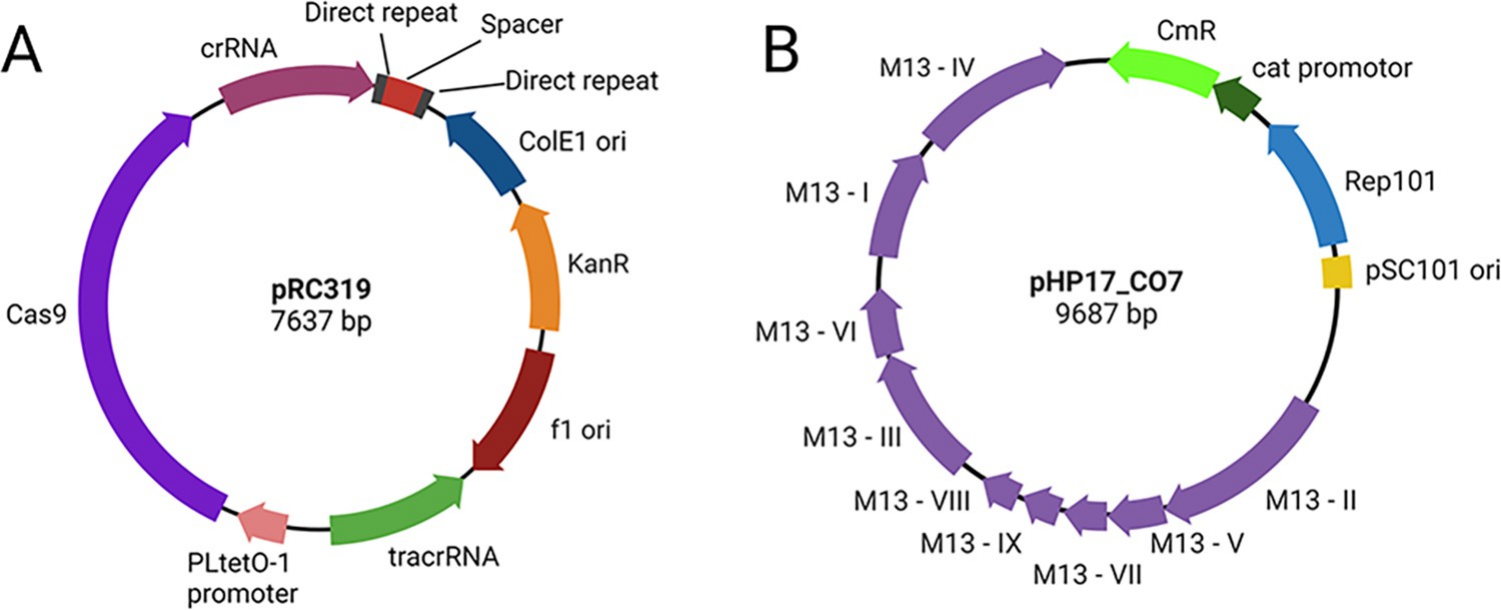
Plasmid map of phagemid and helper phage for phage particle production. (**A**) Phagemid pRC319 containing modified CRISPR-Cas9 targeting *bla*_CTX-M_. (**B**) Modified helper phage pHP17_CO7 containing Cm^R^ gene.

Re-sensitization activity of the programmed CRISPR-Cas9 on phagemid was first evaluated by transformation into the target strains (**Fig 2B**). Each pRC319-G1_I and pRC319-G1_II was transformed into *E*. *coli* K-12: *bla*_CTX-M-15_ and *E*. *coli* K-12: *bla*_CTX-M-55_. pRC319-G9 was transformed into *E*. *coli* K-12: *bla*_CTX-M-14_, *E*. *coli* K-12: *bla*_CTX-M-27_, *E*. *coli* K-12: *bla*_CTX-M-65_, and *E*. *coli* K-12: *bla*_CTX-M-90_. Re-sensitization by pRC319-P targeting the promoter was examined by transformation into *E*. *coli* K-12 carrying each *bla*_CTX-M_ variant. The co-transformants (**Table 1**) in 100-μL volume were recovered by adding SOC medium (1:10 dilution), incubated at 37°C with 200 rpm agitation for 90 min, and then culturing (1:100 dilution) in LB broth containing Km35, incubated for 16 h. Ten-fold serial dilutions of co-transformant were plated onto three selective agar plates that contained (1) Km35, (2) Km35+CTX2, and (3) Km35+Gm15. All plates had 0.2 μg/mL of anhydrotetracycline (aTC) for induction of Cas9 expression. Colonies were enumerated after 16-h incubation. The re-sensitization ratio and plasmid clearance ratio were calculated from averaged colony-forming units (CFU) from three independent biological replicates. Clearance of *bla*_CTX-M_ and presence of pRC319-G1_I, pRC319-G1_II, pRC319-G9, and pRC319-P were detected by PCR from colonies grown on Km35 plates. The modified pRC319 containing CRISPR-Cas9 **(Fig 3A)** that presented re-sensitization activity were included for phage particle (ΦRC319) production with helper phage pHP17_CO7 **(Fig 3B)**.

### Production of ΦRC319 for delivering CRISPR-Cas9

**Fig 4:**
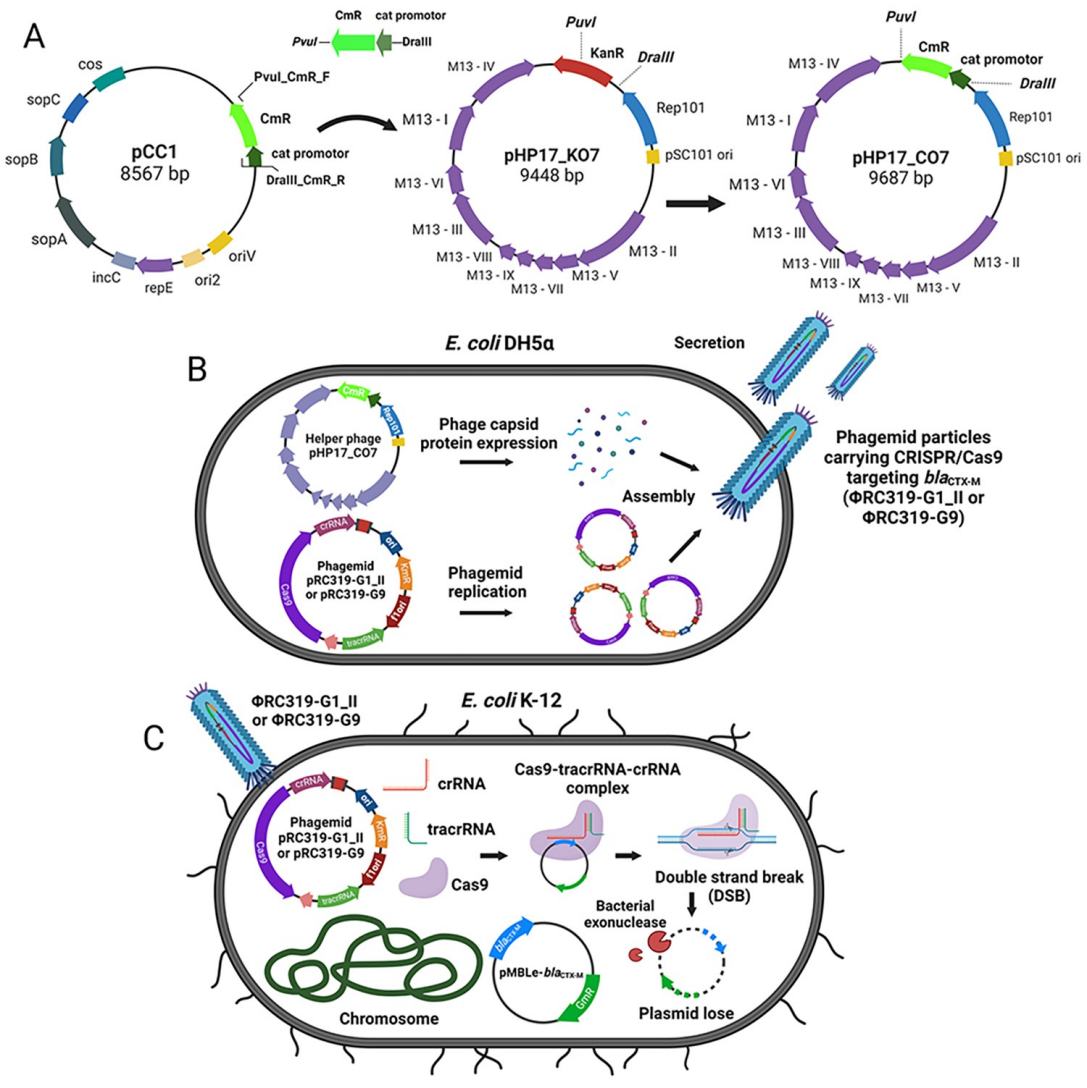
Schematic presenting workflow of phage particle production and antimicrobial re-sensitization by transduction. (**A**) Construction of modified helper phage pHP17_CO7. (**B**) Phage ΦRC319-G1_II and ΦRC319-G9 were produced from co-transformation of helper phage pHP17_CO7 together with pRC319-G1_II or pRC319-G9. (**C**) Phage ΦRC319-G1_II and ΦRC319-G9 contained the programmed CRISPR-Cas9 system in the genome was delivered to *E*. *coli* K-12 that carrying *bla*_CTX-M_ on pMBLe plasmid by transduction. The CRISPR-Cas9 expressed functional Cas9-tracrRNA-crRNA complex to cleave pMBLe-*bla*_CTX-M_ plasmid at the target site that resulted in antimicrobial re-sensitization and plasmid clearance. The figure was generated by BioRender (www.app.biorender.com).

pHP17_CO7 (helper phage) was modified from pHP17_KO7 [35], bearing a kanamycin resistance (Km^R^) selective marker, by inserting a chloramphenicol resistance (Cm^R^) gene that interrupted the Km^R^ gene (**Fig 4A**). The Cm^R^ gene and its promoter (763 bp) were amplified from pCCI by PCR with primers (PvuI_CmR_F and DraIII_CmR_R) and were cloned into pT&A vector before transformation into *E*. *coli* DH5α [36]. The colonies were selected by Am100 and chloramphenicol (12.5 μg/mL; abbreviated to Cm12.5) and confirmed by PCR. pHP17_KO7 and pT&A-Cm^R^ were separately digested by *Pvu*I and *Dra*III, and Cm^R^ gene fragment was ligated to the pHP17_KO7 backbone to generate pHP17_CO7 using T4 ligase. The ligated products were transformed into *E*. *coli* DH5α followed by screening using PCR.

Each pRC319-G1_II and pRC319-G9 transformed into *E*. *coli* DH5α containing pHP17_CO7 to generate co-transformant *E*. *coli* DH5α: pRC319-G1_II: pHP17_CO7 and *E*. *coli* DH5α: pRC319-G9: pHP17_CO7, respectively (**Fig 4B**). The co-transformants were selected using Km15 and Cm12.5 in LB agar and confirmed by PCR to detect each programmed CRISPR-Cas9 in phagemid pRC319 using primers Spc_PspXI_F and pCRISPR_G1_DT_R or pCRISPR_G9_DT_R and pHP17_CO7 using primers PvuI_CmR_F and DraIII_CmR_R.

Phage ΦRC319-G1_II and ΦRC319-G9 particles were produced from overnight-grown culture of the co-transformant *E*. *coli* DH5α in 2 L of 2× YT medium containing Km15 and Cm12.5. After incubation at 37°C with 200 rpm agitation for 18 h and removal of bacterial cells, phage particles were precipitated by PEG/NaCl at 4°C for 24 h, centrifuged at 12,000 *xg* for 20 min, and resuspended in 20 mL SM buffer before filtration by using a 0.45-μM filter membrane. The particles in filtrate were repeatedly precipitated, centrifuged, and suspended for purification [37]. Ten-fold dilutions of phage in SM buffer from 10^−1^ to 10^−8^ were prepared for phage titration by adding 5 mL of the phage diluents into 10 mL of mid-log phage *E*. *coli* K-12 (OD_600_~1.5–2.0). Transductants were selectively grown and enumerated on LB agar containing Km15, and phage titers were calculated to be transductant forming unit per mL (TFU/mL) [38].

### Phage delivering CRISPR-Cas9 for re-sensitization

Each ΦRC319-G1_II and ΦRC319-G9 was inoculated in 200-μL of LB broth containing each target *E*. *coli* K-12: *bla*_CTX-M_ strain (10^8^ CFU/mL) at different multiplicity of infection (MOI) at 0.1, 1, 10, 20, 50, and 100 (**Fig 4C**). ΦRC319-G1_II was added to inoculum of *E*. *coli* K-12: *bla*_CTX-15_ and *E*. *coli* K-12: *bla*_CTX-55_. ΦRC319-G9 was added to inoculum of *E*. *coli* K-12: *bla*_CTX-14_, *E*. *coli* K-12: *bla*_CTX-27_, *E*. *coli* K-12: *bla*_CTX-65_, and *E*. *coli* K-12: *bla*_CTX-90_. Unmodified ΦRC319 and SM buffer were used as controls. After incubation at 37°C for 2 h for phage transduction, inoculum was 10-fold serially diluted with PBS and spotted on two selective aTC-containing LB agar plates including (1) CTX2 and (2) Gm15. Viable CTX^R^ and Gm^R^ cells were counted after incubation at 37°C for 8 h, and reduction was calculated to CFU/mL by comparison with the control.

## Results

### Target sequences on *bla*_CTX-M_ for CRISPR spacer construction

One target sequence at the promoter region of *bla*_CTX-M_ having 25% G+C content was found. Multiple target sequences were presented on alignment of the entire *bla*_CTX-M-15 and -55_ (group 1) and *bla*_CTX-M-14, -27, -65, and -90_ (group 9) sequence. The candidates from each group were first selected based on the highest efficiency score that met the criteria consisting of number of off-targets, base-pairing affinity, base mismatches, self-complementary regions, and G+C content. Due to low ability for re-sensitization of the first candidate sequence of *bla*_CTX-M_ group 1, the second candidate was therefore selected for *bla*_CTX-M_ group 1. All 20-bp target sequences were adjacent to the PAM sequence and had no DNA polymorphism, compared to the tested variants and the promoter (**S1 Fig**). The DNA sequence of the targets and their characteristics were presented in **Table 2**. Aligned with the sequence of all variants in each *bla*_CTX-M_ group in the NCBI Reference Gene Catalog, the candidate target sequence of *bla*_CTX-M_ group 1 (*bla*_CTX-M_ group 1_II) and *bla*_CTX-M_ group 9 were present in 107 of 108 variants (99.07%) of *bla*_CTX-M_ group 9 and 69 of 71 variants (97.18%) of *bla*_CTX-M_ group 9.

**Table 2:** Characteristics of target nucleotide sequence selected from *bla*_CTX-M_ group 1, group 9, and their promoter.

Target	Target sequence (5’-3’)	Protospacer adjacent motif (PAM)	Target on DNA strand (Position)	%G+C content	Efficiency	Off-target
*bla*_CTX-M_ group 1_I	GCTACAGTACAGCGATAACGTGG	TGG	+ (387 to 409)	50%	72.30	Not found
*bla*_CTX-M_ group 1_II	CGAGGTGAAGTGGTATCACGCGG	CGG	−(538 to 560)	55%	71.95	Not found
*bla*_CTX-M_ group 9	ATTGTCGCTGTACTGCAACGCGG	CGG	− (383 to 405)	50%	75.53	Not found
*bla*_CTX-M_ promoter	TATCAAAAATGATTGAAAGGTGG	TGG	+	25%	62.72	Not found

### Target sequences in CRISPR array of pCRISPR and phagemid pRC319

The 341-bp fragments of CRISPR array including inserted oligos were amplified from pCRISPR-G1_I, pCRISPR-G1_II, pCRISPR-G9, and pCRISPR-P, and the nucleotide sequencing revealed presence of the candidate target sequence as a spacer in each modified CRISPR array for expression to be crRNA (**S2 Fig**). After cloning into pRC319, 421-bp fragments of CRISPR region and array were amplified from pRC319-G1_I, pRC319-G1_II, pRC319-G9, and pRC319-P followed by sequencing that revealed the presence of spacer that was identical to those found in the modified pCRISPR.

### Antimicrobial re-sensitization by transformation

**Fig 5:**
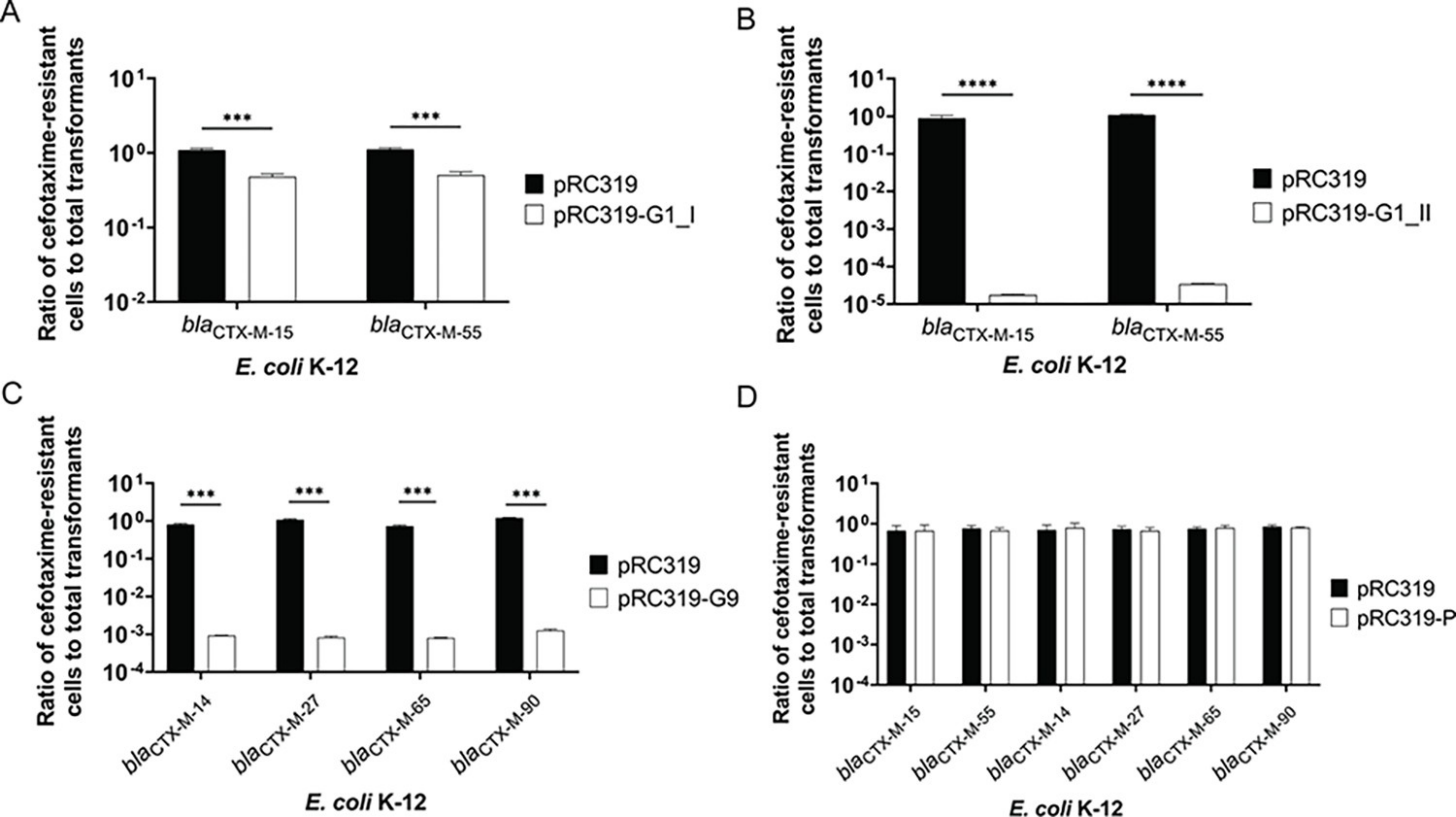
Re-sensitization to cefotaxime (CTX) of *E*. *coli* K-12 carrying *bla*_CTX-M_ on pMBLe (*E*. *coli* K-12: *bla*_CTX-M_) by transformation. Colony-forming units (CFUs) of CTX-resistant (CTX^R^) *E*. *coli* K-12 was enumerated to calculate ratio of viable cells after re-sensitization to total transformants. Reduction of the ratio in treated groups acquiring programmed CRISPR-Cas9-containing pRC319 (white bar) compared with ratio (approximately 1) in the control groups acquiring unmodified pRC319 (black bar). (**A**) Ratio of CTX^R^ cells to total transformants of *E*. *coli* K-12: *bla*_CTX-M-15_: pRC319-G1_I and *E*. *coli* K-12: *bla*_CTX-M-55_: pRC319-G1_I. (**B**) Ratio of CTX^R^ cells to total transformants of *E*. *coli* K-12: *bla*_CTX-M-15_: pRC319-G1_II and *E*. *coli* K-12: *bla*_CTX-M-55_: pRC319-G1_II. (**C**) Ratio of CTX^R^ cells to total transformants of *E*. *coli* K-12: *bla*_CTX-M-14_: pRC319-G9, *E*. *coli* K-12: *bla*_CTX-M-27_: pRC319-G9, *E*. *coli* K-12: *bla*_CTX-M-65_: pRC319-G9, and *E*. *coli* K-12: *bla*_CTX-M-90_: pRC319-G9. (**D**) Ratio of CTX^R^ cells to total transformants of *E*. *coli* K-12 carrying *bla*_CTX-M_ group 1: pRC319-P and *bla*_CTX-M_ group 9: pRC319-P. Error bars represent results from three biological replicates. **P<0.01 was considered statistically significant that was calculated using the Student’s t-test, and bar charts were generated using GraphPad Prism version 8.0.

**Fig 6:**
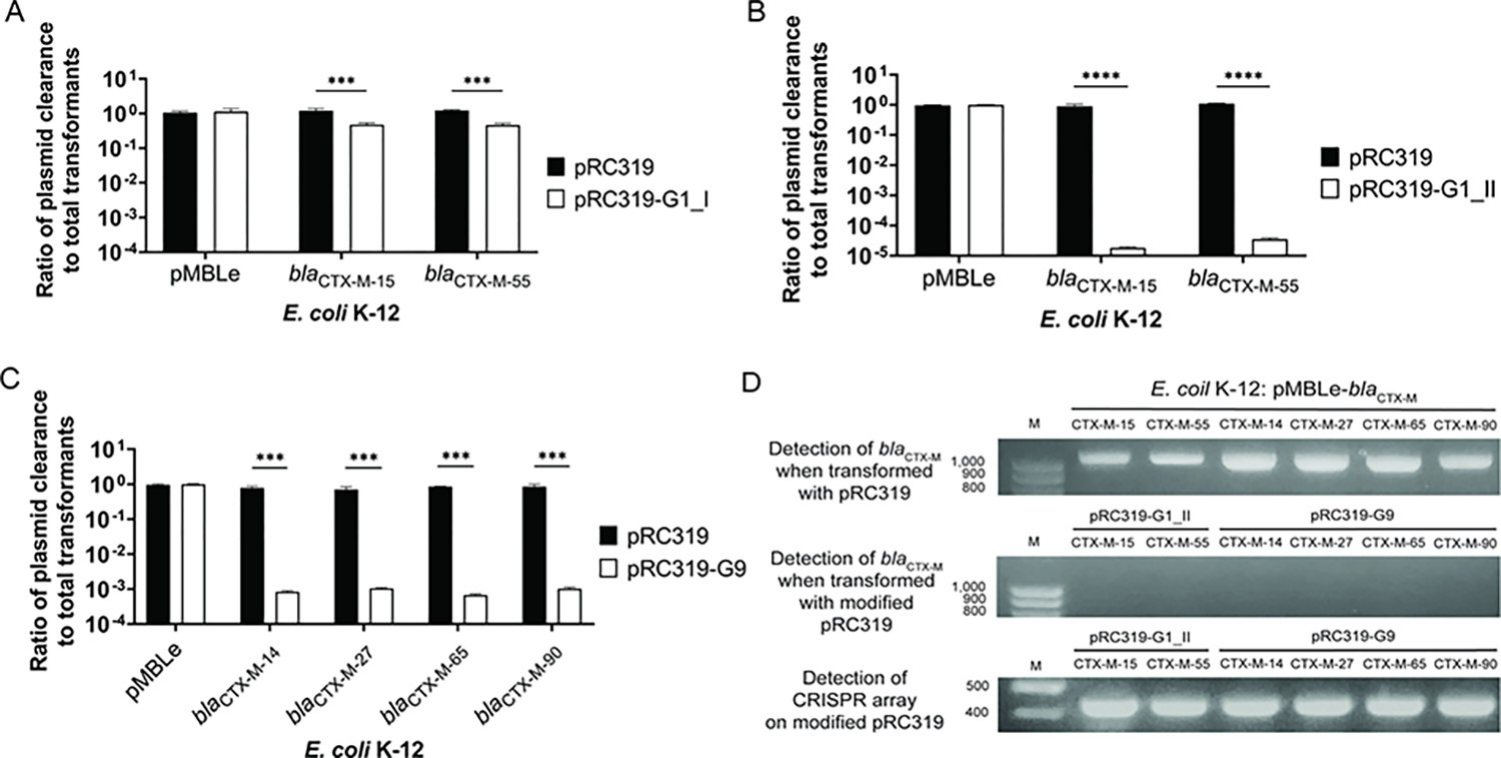
Clearance of plasmid pMBLe: *bla*_CTX-M_ in *E*. *coli* K-12 carrying *bla*_CTX-M_ on pMBLe (*E*. *coli* K-12: *bla*_CTX-M_). Colony-forming units (CFUs) of gentamicin-resistant (Gm^R^) *E*. *coli* K-12 was enumerated to calculate ratio of viable cells after plasmid clearance to total transformant. Reduction of the ratio in treated groups acquiring programmed CRISPR-Cas9-containing pRC319 (white bar) compared with ratio (approximately 1) in the control groups acquiring unmodified pRC319 (black bar). (**A**) Ratio of CTX^R^ cells to total transformants of *E*. *coli* K-12: pMBLe: pRC319-G1_I, *E*. *coli* K-12: *bla*_CTX-M-15_: pRC319-G1_I, and *E*. *coli* K-12: *bla*_CTX-M-55_: pRC319-G1_I. (**B**) Ratio of CTX^R^ cells to total transformants of *E*. *coli* K-12: pMBLe: pRC319-G1_II, *E*. *coli* K-12: *bla*_CTX-M-15_: pRC319-G1_II, and *E*. *coli* K-12: *bla*_CTX-M-55_: pRC319-G1_II. (**C**) Ratio of CTX^R^ cells to total transformants of *E*. *coli* K-12: pMBLe: pRC319-G9, *E*. *coli* K-12: *bla*_CTX-M-14_: pRC319-G9, *E*. *coli* K-12: *bla*_CTX-M-27_: pRC319-G9, *E*. *coli* K-12: *bla*_CTX-M-65_: pRC319-G9, and *E*. *coli* K-12: *bla*_CTX-M-90_: pRC319-G9. (**D**) Amplicon of *bla*_CTX-M_ gene (1,100 bp) and CRISPR array (421 bp) amplified by colony PCR, the *bla*_CTX-M_ amplicon was detected from pMBLe-*bla*_CTX-M_-carrying *E*. *coli* K-12 without modified pRC319 transformation but no amplification of *bla*_CTX-M_ in each group with the modified pRC319 transformation. Moreover, CRISPR array from the modified pRC319 was detected in re-sensitized cells indicated successful transformation of modified pRC319. Error bars represent results from three biological replicates. **P<0.01 was considered statistically significant that was calculated using the Student’s t-test, and bar charts were generated using GraphPad Prism version 8.0.

Re-sensitization activity was present in the programmed CRISPR-Cas9 that targeted internal sequence of *bla*_CTX-M_ group 1 and *bla*_CTX-M_ group 9 but was not found in that targeting the promoter region. Viable cells were not reduced in controls of all *E*. *coli* K-12: *bla*_CTX-M_ strains treated with pRC319 transformation that resulted in a ratio of viable cells of 1. In the *bla*_CTX-M_ group 1, the transformants *E*. *coli* K-12: *bla*_CTX-M-15_: pRC319-G1_I and *E*. *coli* K-12: *bla*_CTX-M-55_: pRC319-G1_I exhibited a 0.5 log_10_ reduction in ratios (**Fig 5A**), whereas *E*. *coli* K-12: *bla*_CTX-M-15_: pRC319-G1_II and *E*. *coli* K-12: *bla*_CTX-M-55_: pRC319-G1_II displayed a 4 log_10_ reduction (**Fig 5B**), indicating enhanced re-sensitization activity. For *bla*_CTX-M_ group 9, re-sensitization in *E*. *coli* K-12: *bla*_CTX-M-14_: pRC319-G9, *E*. *coli* K-12: *bla*_CTX-M-27_: pRC319-G9, *E*. *coli* K-12: *bla*_CTX-M-65_: pRC319-G9, and *E*. *coli* K-12: *bla*_CTX-M-90_: pRC319-G9 resulted in decreasing approximately 3 log_10_ of ratios of viable cells (**Fig 5C**). Absence of re-sensitization activity was observed in *E*. *coli* K-12 carrying *bla*_CTX-M_ group 1: pRC319-P and *bla*_CTX-M_ group 9: pRC319-P (**Fig 5D**). Besides, plasmid clearance activity was presented in the groups treated with pRC319-G1_I, pRC319-G1_II, and pRC319-G9 transformation (**Fig 6A, 6B, and 6C**). Negative *bla*_CTX-M_ and positive CRISPR array amplification from colonies of re-sensitized cells confirmed the successful transformation of the modified phagemid and cleavage of *bla*_CTX-M_ gene, as presented in **Fig 6D**. Therefore, pRC319-G1_II and pRC319-G9 that show high re-sensitization efficiency were used for phage particle production.

### Phage particles containing the CRISPR-Cas9 targeting *bla*_CTX-M_

pRC319-G1_II and pRC319-G9 were included for phage particle production due to their high re-sensitization efficiency, whereas pRC319-G1_I and pRC319-P were excluded due to the low re-sensitization efficiency and the absence of re-sensitization, respectively. Co-transformant *E*. *coli* DH5α: pRC319: pHP17_CO7, *E*. *coli* DH5α: pRC319-G1_II: pHP17_CO7, and *E*. *coli* DH5α: pRC319-G9: pHP17_CO7 successfully grew on LB agar supplemented with Km35 and Cm12.5 (**S3A Fig**). Colony PCR detected positive specific amplicons of pHP17_CO7 (842 bp) and CRISPR array in the modified pRC319-G1_II and pRC319-G9 (421 bp) (**S3B Fig**). Titration of phage ΦRC319, ΦRC319-G1_II, and ΦRC319-G9 that were produced from overnight culture of the co-transformant showed 2.4×10^10^ TFU/mL, 2.20×10^10^ TFU/mL, and 2.36×10^10^ TFU/mL, respectively.

### Antimicrobial re-sensitization by phage delivering the programmed CRISPR-Cas9 system

**Fig 7:**
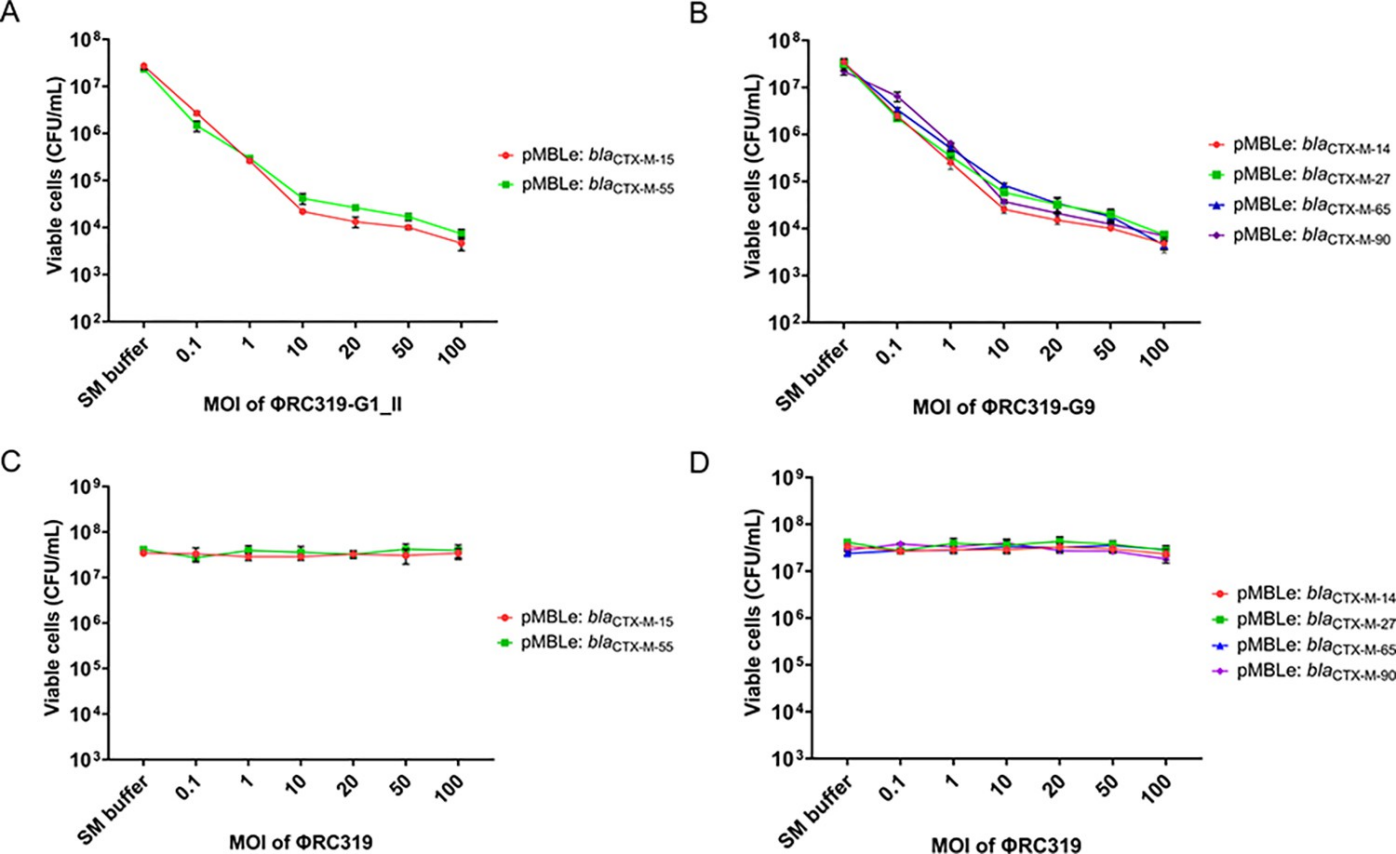
Dose-dependent reduction of viable cefotaxime-resistant (CTX^R^) *E*. *coli* K-12 carrying *bla*_CTX-M_ on pMBLe (*E*. *coli* K-12: *bla*_CTX-M_) after re-sensitization by the programmed CRISPR-Cas9 delivered by phage transduction at multiplicity of infection (MOI) from 0.1 to 100. (**A**) Viable CTX^R^ cells of transductant *E*. *coli* K-12: *bla*_CTX-M-15_: ΦRC319-G1_II and *E*. *coli* K-12: *bla*_CTX-M-55_: ΦRC319-G1_II. (**B**) Viable CTX^R^ cells of transductant *E*. *coli* K-12: *bla*_CTX-M-14_: ΦRC319-G9, *E*. *coli* K-12: *bla*_CTX-M-27_: ΦRC319-G9, *E*. *coli* K-12: *bla*_CTX-M-65_: ΦRC319-G9, and *E*. *coli* K-12: *bla*_CTX-M-90_: ΦRC319-G9. (**C**) Viable CTX^R^ cells of transductant *E*. *coli* K-12: *bla*_CTX-M-15_: ΦRC319 and *E*. *coli* K-12: *bla*_CTX-M-55_: ΦRC319 (control group). (**D**) Viable CTX^R^ cells of transductant *E*. *coli* K-12: *bla*_CTX-M-14_: ΦRC319, *E*. *coli* K-12: *bla*_CTX-M-27_: ΦRC319, *E*. *coli* K-12: *bla*_CTX-M-65_: ΦRC319, and *E*. *coli* K-12: *bla*_CTX-M-90_: ΦRC319 (control group). Error bars represent results from three biological replicates. The line chart was generated using GraphPad Prism version 8.0.

ΦRC319-G1_II and ΦRC319-G9 delivering the programmed CRISPR-Cas9 by transduction presented dose-dependent reduction of viable cells from 1 log_10_ to 4 log_10_ of transductant *E*. *coli* K-12: *bla*_CTX-M-15_: ΦRC319-G1_II, *E*. *coli* K-12: *bla*_CTX-M-55_: ΦRC319-G1_II, *E*. *coli* K-12: *bla*_CTX-M-14_: ΦRC319-G9, *E*. *coli* K-12: *bla*_CTX-M-27_: ΦRC319-G9, *E*. *coli* K-12: *bla*_CTX-M-65_: ΦRC319-G9, and *E*. *coli* K-12: *bla*_CTX-M-90_: ΦRC319-G9 when increasing MOI from 0.1 to 100 (**Fig 7A and 7B**). In control groups, reduction was not detected after treatment with ΦRC319 (**Fig 7C and 7D**). At MOI 100, the highest re-sensitization efficacy was presented by 4 log_10_ reduction of the viable cells that could grow on agar with the presence of CTX. Plasmid clearance activity was also presented in a similar reduction ratio to that of re-sensitization activity. Plasmid clearance activity data may not be shown.

## Discussion

Various studies present the ability of programmable CRISPR-Cas9 system for the reversal of AMR bacteria that have corresponding resistance mechanisms mediated by specific antimicrobial resistance genes to be susceptible to the antimicrobials. Our results proposed searching of common candidate sequences for programming CRISPR-Cas9 by construction of spacer to be a programmed Cas9-tracrRNA-crRNA complex that breakdown multiple variants of major *bla*_CTX-M_. Regarding high variation of *bla*_CTX-M_, only re-sensitization by CRISPR-Cas9 catalyzing most widespread *bla*_CTX-M-15_ was studied [24]. However, it was not possible to find consensus regions shared between *bla*_CTX-M_ groups to be candidate spacers because of low nucleotide similarity and lack of adjacent PAM sequence. Therefore, we selected candidate target sequences from each *bla*_CTX-M_ group that were capable of programming a spectrum of CRISPR-Cas9 functions. Consistent re-sensitization activity of CRISPR-Cas9 among tested variants in each group supported this approach. Promoter or transcription initiation regions of antimicrobial resistance genes are other targets for of CRISPR-Cas9 system for re-sensitization or gene silencing in bacteria and mammalian cells [22, 39]. Knocking down expression of ADP-ribosyl transferase using CRISPRi-dCas9 targeting the promoter of *arr* gene exploits successful re-sensitization of *Mycobacterium smegmatis* to be susceptible to rifampicin [22]. The promoter of *bla*_CTX-M_, commonly located on the IS*Ecp1*, was selected to be a broad target of *bla*_CTX-M_ in this study. However, effective re-sensitization was not obtained by targeting this region. Because of the narrow range and presence of only one PAM sequence, the promoter is not the good target for *bla*_CTX-M_ inactivation. Therefore, the internal sequence of *bla*_CTX-M_ gene that had a variety of region adjacent to PAM sequences had a possibility for searching good candidate target sequences for CRISPR-Cas9.

Re-sensitization activity was observed in the programmed CRISPR-Cas9 with the highest efficacy, the second candidate target sequence of *bla*_CTX-M_ group 1 and that of *bla*_CTX-M_ group 9 can be found in up to 99% (107 of 108 variants) and 97% (69 of 71 variants) of the *bla*_CTX-M_ variants in each group, respectively [6]. *In vitro* efficacy was examined for the prevalent variants that were available for plasmid construction, and further variants should be further evaluated. Target sequence selection for CRISPR construction and production of guide RNA (gRNA) is a critical step for CRISPR-Cas9 re-sensitization efficacy. For *bla*_CTX-M_ genes, gene classification and epidemiological information are useful for target sequence selection due to the absence of candidate consensus sequence of all *bla*_CTX-M_ variants. In contrast, common target sequences for CRISPR-Cas9 on *bla*_TEM_ and *bla*_SHV_, which also have high numbers of variants are detected, and selected for spacer design that results in clearance of AMR gene-containing plasmid and 99% re-sensitization in *E*. *coli* [21]. In *bla*_CTX-M_ group 1, a new spacer in pRC319-G1_II was selected due to the low re-sensitization efficacy of pRC319-G1_I that might be a result of low gRNA affinity. DNA region adjacent to the target sequence could affect gRNA activity such as polymorphisms in the PAM sequence and flanking regions [40]. 5′-CGG PAM sequence, which was the PAM sequence of pRC319-G1_II and pRC319-G9, had a higher binding affinity to targets than other PAM sequences for CRISPR-Cas9 [41]. Additionally, affinity of “seed sequence”, which is the 10–12 nucleotides adjacent to PAM, strongly affects base pairing of gRNA to the target sequence that is a key role for CRISPR array designation [42]. As presented in target sequence in the *bla*_CTX-M_ promoter, borderline G+C content at 25% might retard the binding affinity between the gRNA and the promoter region that causes incomplete pairing and loss of double strand break activity [43]. However, few spacer sequences were selected for CRISPR construction in this study which was a limitation for comparison of efficacy and specificity. The most effective single spacer for each *bla*_CTX-M_ group cannot re-sensitized all resistant cells that are similar to results from previous works [27]. Improving strategies such as inserting multiple spacers and creating multiple CRISPR-Cas copies could be merged to in CRISPR-Cas9 development [24, 44].

Numbers of re-sensitized cells exponentially increased depending on numbers of phagemid particles that presented CRISPR-Cas9 delivery by the non-replicative phagemid transduction. Bacteriophage has been popularly used as a biological vehicle for introduction of exogenous DNA into bacterial cells. Previously developed phagemid pRC319 contained CRISPR-Cas9 components, multiple recognition site, and f1 origin of replication derived from bacteriophage that fully supported genetic modification, evaluation of CRISPR spacers by transformation, and phagemid particle production in our study. Production of phagemid particle required structural capsid proteins from M13-derivative helper plasmid pHP17_CO7 that was modified from pHP17_KO7, which has kanamycin resistance marker (aminoglycoside phosphotransferase), because of the similar antibiotic resistance selective marker with the modified pRC319, which has neomycin/kanamycin resistance marker. Using co-transformation of pRC319 and pHP17_KO7, very low phage particles were obtained, and loss maintenance of pHP17_KO7 was observed. Phage particle production was achieved by helper plasmid modification to be pHP17_CO7 by insertion of chloramphenicol acetyltransferase gene that supported co-selection of both phagemid and helper plasmid in the co-transformant. In phagemid system, phagemid and helper plasmid containing different antimicrobial resistance genes that need different antimicrobial classes for selection supports bacteria to continuously maintain both in culture of phage particle production system [45]. Because pHP17_CO7 is a low-copy number plasmid and lacks its packaging signal, the modified phagemid genome could be sufficiently packaged to be a CRISPR-Cas9 carrier [35]. However, helper plasmid particles without the modified pRC319 were not investigated. Compatibility of the helper plasmid pHP17_CO7 and the modified pRC319 containing constructed CRISPR-Cas9 system efficiently generated high numbers (10^10^ TFU/mL) of phagemid pRC319 particles in the re-sensitization model.

In this study, we used pCRISPR as an intermediate vector that is adaptable for spacer construction and serves next-step recombination with the phagemid pRC319. This vector is prepared for two delivery modes, including transformation for the evaluation of the constructed CRISPR-Cas system and transduction after phage production. The re-sensitization efficacy was limited only *in vitro* of *E*. *coli* K-12 model in this study. Due to the narrow host range of M13-derived phagemid particle, CRISPR-Cas9 might not be delivered into F-pilus-deficient *E*. *coli* strains that needs strategies to expand host range. Effective re-sensitization was presented by catalytic activity on predominant *bla*_CTX-M_ variants in group 1 and group 9 that were available in our study; however, ability to cleavage other less prevalent variants should be further evaluated. The platform used in our study promoted customizing CRISPR-Cas9 construction to target antimicrobial resistance genes for antimicrobial re-sensitization or to inactivate other genes in *E*. *coli* in one-direction, two-step process. Spacers of *bla*_CTX-M_ genes could be selected from internal sequence that are shared among variants in each group. Epidemiological distribution and AMR gene evolution should be continuously monitored together with the development of gene-based tools combatting AMR. Simplification of cloning methods and development of vector backbone are still promising to facilitate effective introduction of CRISPR-Cas9 and expression in bacterial cells.

## Conclusion

This study demonstrated the re-sensitization by phagemid particle carrying CRISPR-Cas9 system. The modified CRISPR-Cas9 delivered by phagemid particle effectively destroys major *bla*_CTX-M_ variants in *E*. *coli* resulting in double strand break and loss of entire plasmid. Selection of candidate spacers from the conserved regions in *bla*_CTX-M_ groups could serve as a model for broadening cleavage activity of CRISPR-Cas9, targeting not only a single AMR gene, but also those with a high number of variants. Consequently, this technique could be further developed as a promising tool for tackling antimicrobial resistance.

## Supporting information

S1 Fig The candidate target sequences and PAM sequences from the *bla*_CTX-M_ alignment. (**A**) gRNA and PAM sequences for *bla*_CTX-M_ group 1, including the first candidate target sequence (G1_I) and the second candidate target sequence (G1_II). (**B**) gRNA and PAM sequences for *bla*_CTX-M_ group 9 (G9). (**C**) gRNA and PAM sequences for *bla*_CTX-M_ promoter group 1 and 9 (P). The color indications are as follows: Green color, indicating the mutation points; Yellow color, indicating the PAM sequences; Blue color, indicating the gRNA sequences; Underline text, indicating start and stop codons; Black box, indicating the promoter at -35 and -10.
(PDF)

S2 Fig The nucleotide sequence and structure of the CRISPR region of the modified pCRISPR with the presence of inserted target sequence in spacer region. (**A**) pCRISPR-G1_I. (**B**) pCRISPR-G1_II. (**C**) pCRISPR-G9 and (**D**) pCRISPR-P. The inserted spacer between the two repeats (DR) of the CRISPR array is indicated by a red box.
(PDF)

S3 Fig The selection of co-transformant *Escherichia coli* DH5α carrying pHP17_CO7 and modified pRC319 and the detection of both plasmid by colony PCR. (**A**) The growth of *E*. *coli* DH5α carrying pHP17_CO7 and modified pRC319 (on the right) was observed on LB agar containing kanamycin (35 μg/mL) and chloramphenicol (12.5 μg/mL). However, no growth was observed for *E*. *coli* DH5α carrying pHP17_CO7 (on the left). (**B**) A 421-bp amplicon of CRISPR array in the modified pRC319 was obtained (lane 1: pRC319-G1_II; lane 3, pRC319-G9), and an 842-bp amplicon of specific region in pHP17_CO7 was obtained (lane 2 and lane 4) from each co-transformant.
(PDF)

S1 Table Plasmids used in this study. (DOCX)

S2 Table Primers used in this study. (DOCX)

S3 Table Target sequences of the predominant *bla*_CTX-M_ group 1, group 9, and promoter for construction of spacer. (DOCX)
