# Supplementary material for: Non-replicative phage particles delivering CRISPR-Cas9 to target major blaCTX-M variants
Source: PLoS One. 2024 May 16;19(5):e0303555. doi: 10.1371/journal.pone.0303555 (PMC11098365; doi:10.1371/journal.pone.0303555)
Supplement: S1 Fig — (A) gRNA and PAM sequences for blaCTX-M group 1, including the first candidate target sequence (G1_I) and the second candidate target sequence (G1_II). (B) gRNA and PAM sequences for blaCTX-M group 9 (G9). (C) gRNA and PAM sequences for blaCTX-M promoter group 1 and 9 (P). The color indications are as follows: Green color, indicating the mutation points; Yellow color, indicating the PAM sequences; Blue color, indicating the gRNA sequences; Underline text, indicating start and stop codons; Black box, indicating the promoter at -35 and -10. (PDF) [file pone.0303555.s001.pdf]

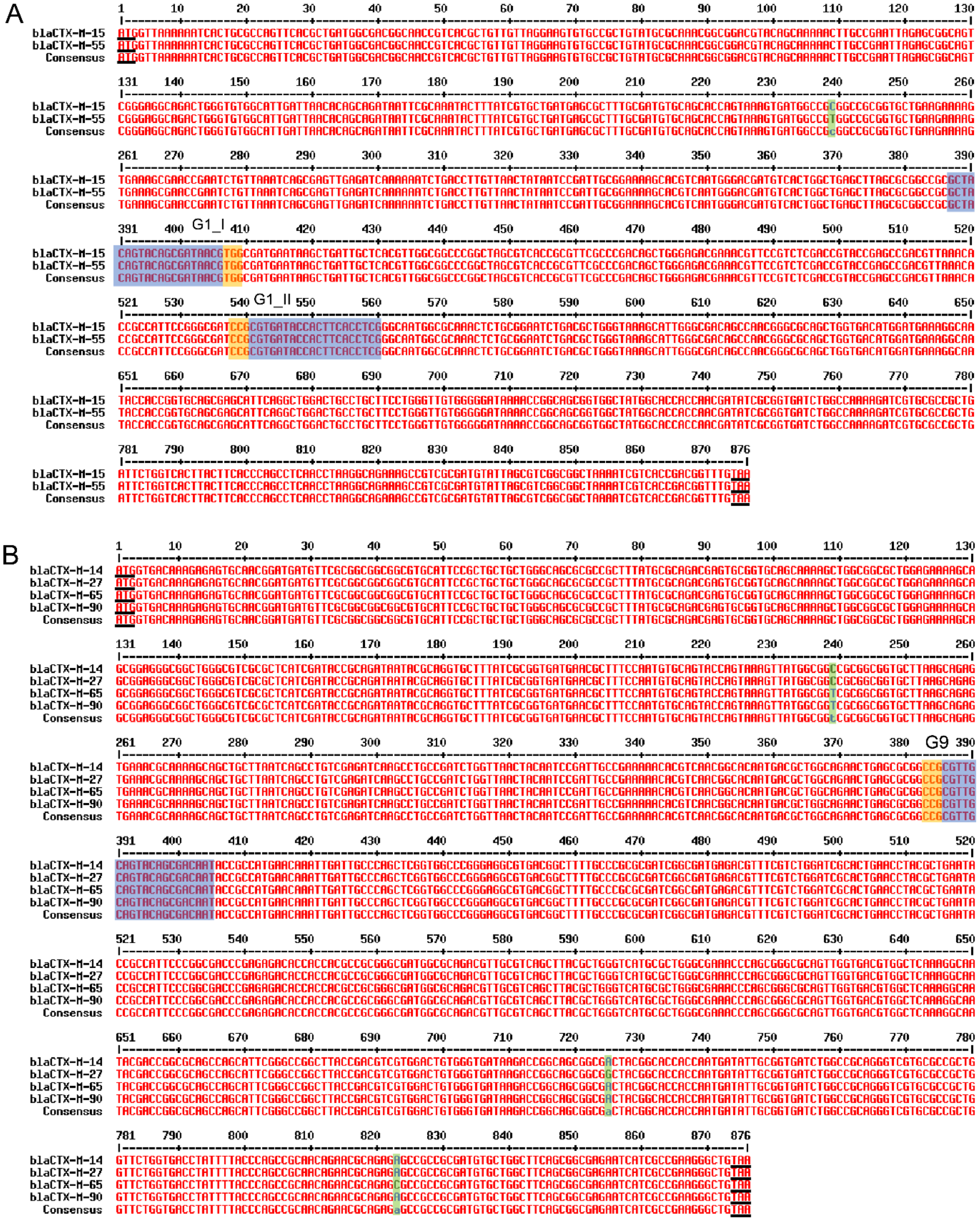
A
G1_I
G1_II
blaCTX-M-15
blaCTX-M-55
Consensus
B
G9
blaCTX-M-14
blaCTX-M-27
blaCTX-M-65
blaCTX-M-90
Consensus

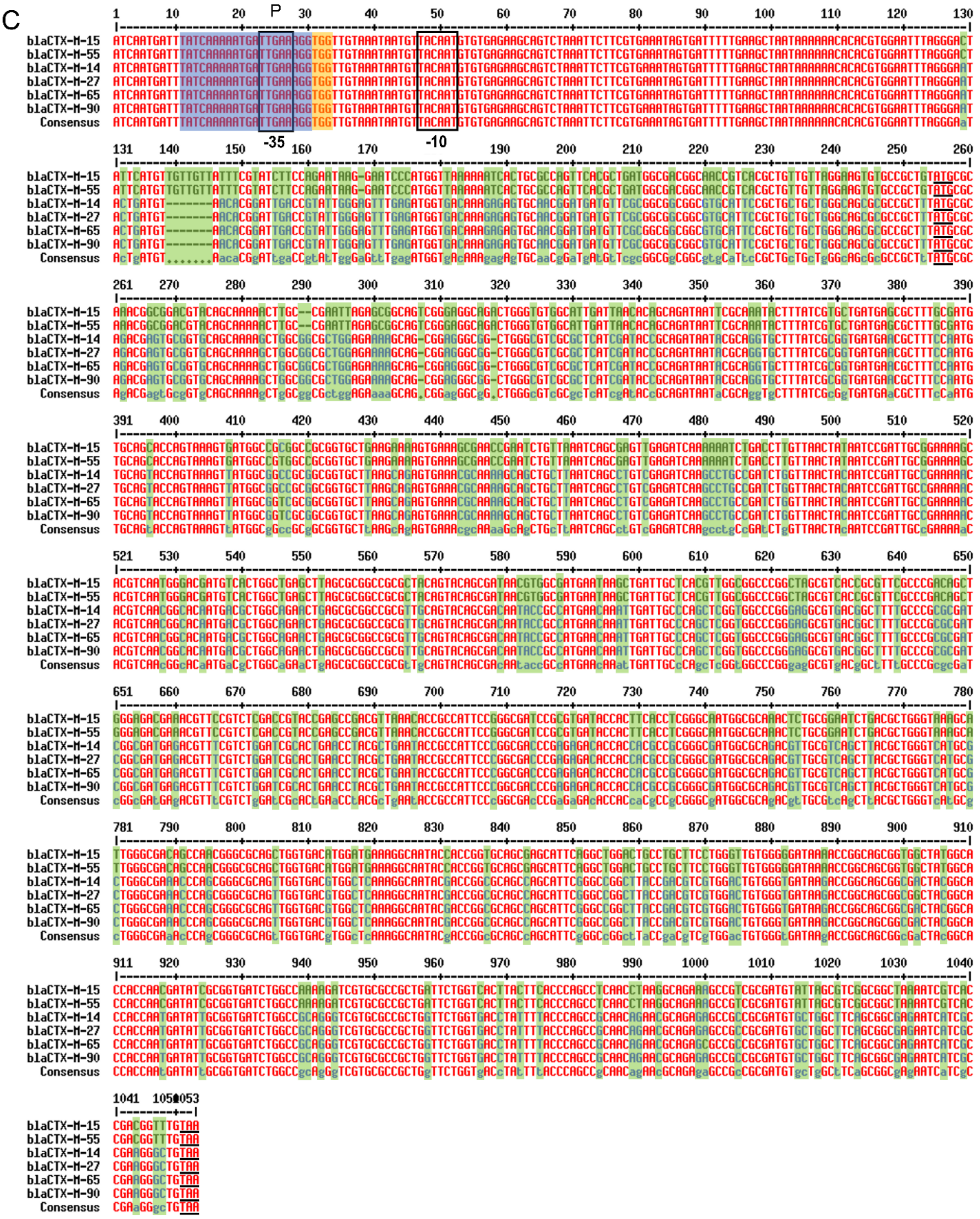

S1 Fig. The candidate target sequences and PAM sequences from the *bla*$_{\text{CTX-M}}$ alignment. (**A**) gRNA and PAM sequences for *bla*$_{\text{CTX-M}}$ group 1, including the first candidate target sequence (G1_I) and the second candidate target sequence (G1_II). (**B**) gRNA and PAM sequences for *bla*$_{\text{CTX-M}}$ group 9 (G9). (**C**) gRNA and PAM sequences for *bla*$_{\text{CTX-M}}$ promoter

group 1 and 9 (P). The color indications are as follows: Green color, indicating the mutation points; Yellow color, indicating the PAM sequences; Blue color, indicating the gRNA sequences; Underline text, indicating start and stop codons; Black box, indicating the promoter at -35 and -10.
