# Supplementary material for: Non-replicative phage particles delivering CRISPR-Cas9 to target major blaCTX-M variants
Source: PLoS One. 2024 May 16;19(5):e0303555. doi: 10.1371/journal.pone.0303555 (PMC11098365; doi:10.1371/journal.pone.0303555)
Supplement: S2 Fig — (A) pCRISPR-G1_I. (B) pCRISPR-G1_II. (C) pCRISPR-G9 and (D) pCRISPR-P. The inserted spacer between the two repeats (DR) of the CRISPR array is indicated by a red box. (PDF) [file pone.0303555.s002.pdf]

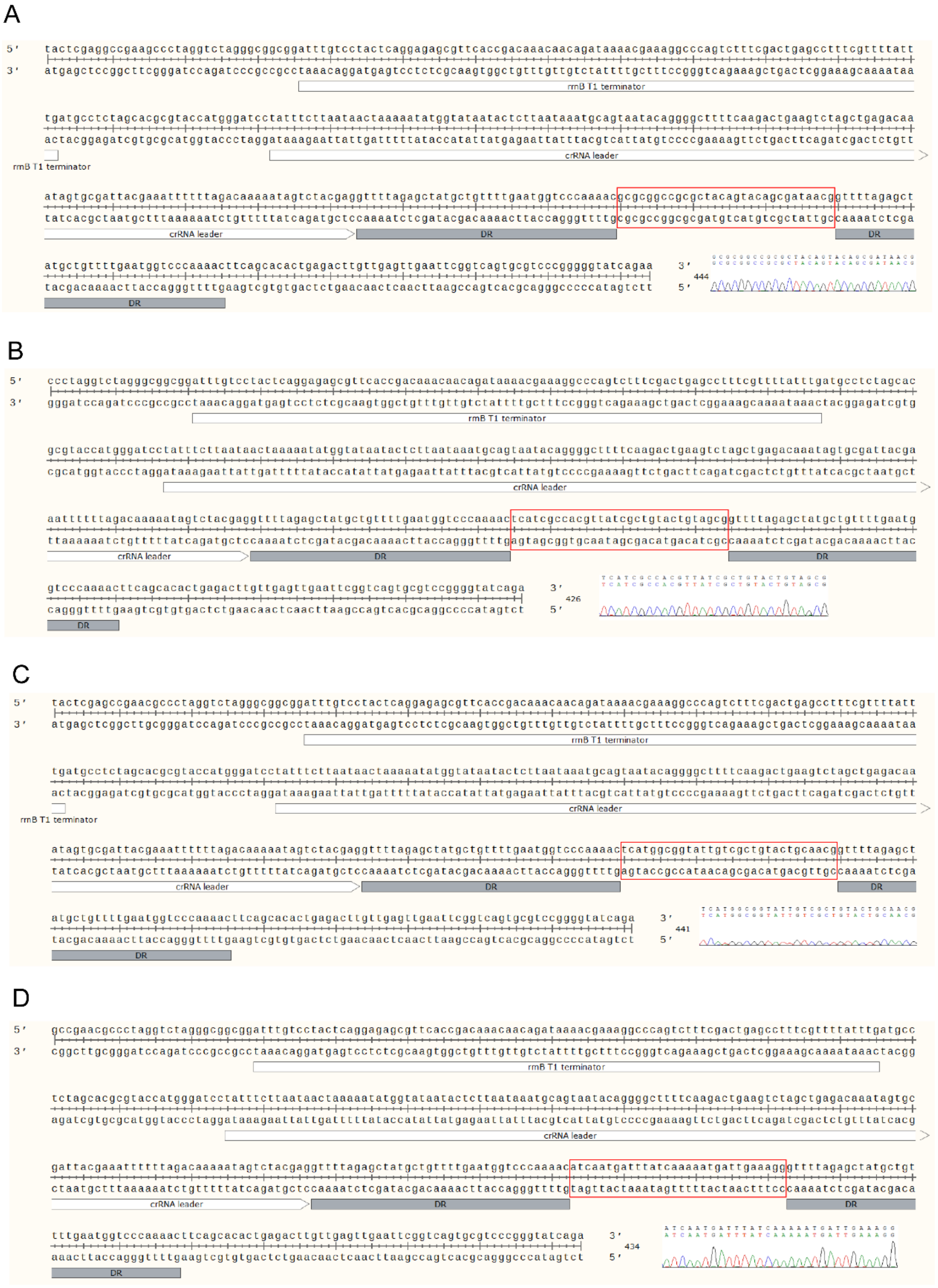

S2 Fig. The nucleotide sequence and structure of the CRISPR region of the modified pCRISPR with the presence of inserted target sequence in spacer region. (A) pCRISPR-

G1_I. (**B**) pCRISPR-G1_II. (**C**) pCRISPR-G9 and (**D**) pCRISPR-P. The inserted spacer between the two repeats (DR) of the CRISPR array is indicated by a red box.
