# Supplementary material for: Non-replicative phage particles delivering CRISPR-Cas9 to target major blaCTX-M variants
Source: PLoS One. 2024 May 16;19(5):e0303555. doi: 10.1371/journal.pone.0303555 (PMC11098365; doi:10.1371/journal.pone.0303555)
Supplement: S3 Fig — (A) The growth of E. coli DH5α carrying pHP17_CO7 and modified pRC319 (on the right) was observed on LB agar containing kanamycin (35 μg/mL) and chloramphenicol (12.5 μg/mL). However, no growth was observed for E. coli DH5α carrying pHP17_CO7 (on the left). (B) A 421-bp amplicon of CRISPR array in the modified pRC319 was obtained (lane 1: pRC319-G1_II; lane 3, pRC319-G9), and an 842-bp amplicon of specific region in pHP17_CO7 was obtained (lane 2 and lane 4) from each co-transformant. (PDF) [file pone.0303555.s003.pdf]

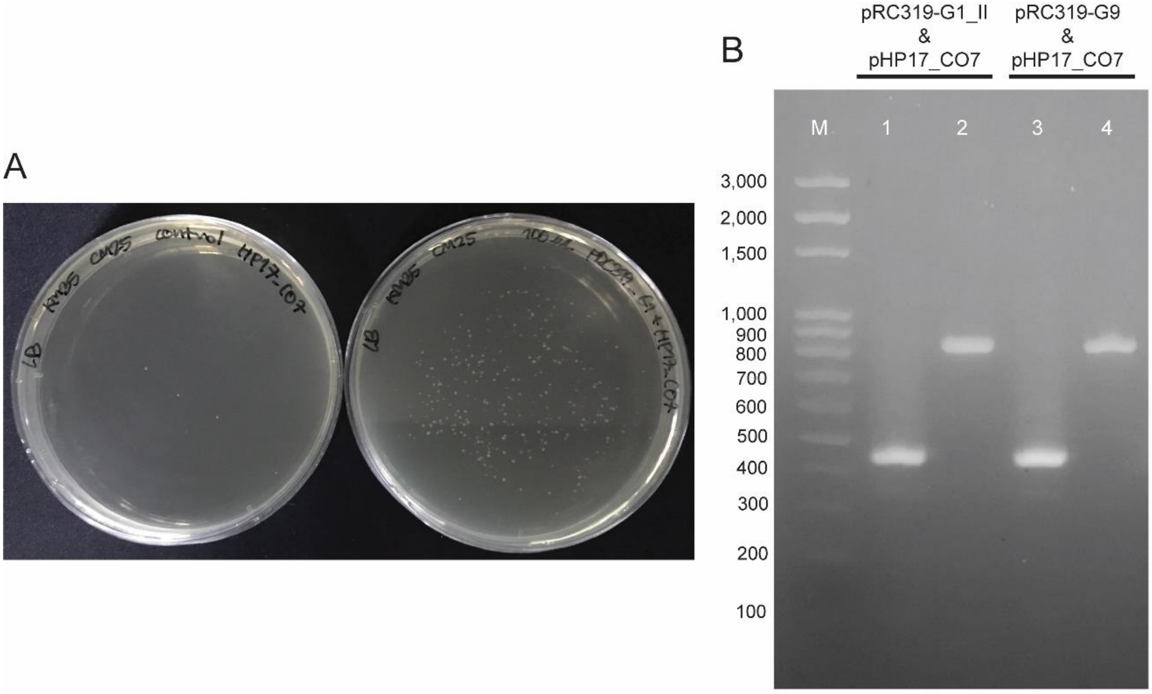

S3 Fig. The selection of co-transformant *Escherichia coli* DH5α carrying pHP17_CO7 and modified pRC319 and the detection of both plasmid by colony PCR. (**A**) The growth of *E. coli* DH5α carrying pHP17_CO7 and modified pRC319 (on the right) was observed on LB agar containing kanamycin (35 µg/mL) and chloramphenicol (12.5 µg/mL). However, no growth was observed for *E. coli* DH5α carrying pHP17_CO7 (on the left). (**B**) A 421-bp amplicon of CRISPR array in the modified pRC319 was obtained (lane 1: pRC319-G1_II; lane 3, pRC319-G9), and an 842-bp amplicon of specific region in pHP17_CO7 was obtained (lane 2 and lane 4) from each co-transformant.
