# Supplementary material for: Non-replicative phage particles delivering CRISPR-Cas9 to target major blaCTX-M variants
Source: PLoS One. 2024 May 16;19(5):e0303555. doi: 10.1371/journal.pone.0303555 (PMC11098365; doi:10.1371/journal.pone.0303555)
Supplement: S1 Table — (DOCX) [file pone.0303555.s004.docx]

**S1 Table. Plasmids used in this study.**

| **Plasmid name** | **Selective marker** | **Relevant features** | **Reference** |
| --- | --- | --- | --- |
| pMBLe | Gm^R^ | *bla*_CTX-M_ expression vector | [29] |
| pMBLe-*bla*_CTX-M-15_ | Gm^R^ and CTX^R^ | *bla*_CTX-M-15_ expression vector | This study |
| pMBLe-*bla*_CTX-M-55_ | Gm^R^ and CTX^R^ | *bla*_CTX-M-55_ expression vector | This study |
| pMBLe-*bla*_CTX-M-14_ | Gm^R^ and CTX^R^ | *bla*_CTX-M-14_ expression vector | This study |
| pMBLe-*bla*_CTX-M-27_ | Gm^R^ and CTX^R^ | *bla*_CTX-M-27_ expression vector | This study |
| pMBLe-*bla*_CTX-M-65_ | Gm^R^ and CTX^R^ | *bla*_CTX-M-65_ expression vector | This study |
| pMBLe-*bla*_CTX-M-90_ | Gm^R^ and CTX^R^ | *bla*_CTX-M-90_ expression vector | This study |
| pCRISPR (Plasmid #42875) | Neo^R^/Km^R^ | Spacer construction | [34] |
| pCRISPR-G1_I | Neo^R^/Km^R^ | Spacer targeting *bla*_CTX-M_ group 1 target I | This study |
| pCRISPR-G1_II | Neo^R^/Km^R^ | Spacer targeting *bla*_CTX-M_ group 1 target II | This study |
| pCRISPR-G9 | Neo^R^/Km^R^ | Spacer targeting *bla*_CTX-M_ group 9 | This study |
| pCRISPR-P | Neo^R^/Km^R^ | Spacer targeting *bla*_CTX-M_ promoter | This study |
| pRC319 (Plasmid #61272) | Neo^R^/Km^R^ | Phagemid carrying CRISPR-Cas9 system | [27] |
| pRC319-G1_I | Neo^R^/Km^R^ | Phagemid carrying CRISPR-Cas9 targeting *bla*_CTX-M_ group 1 target I | This study |
| pRC319-G1_II | Neo^R^/Km^R^ | Phagemid carrying CRISPR-Cas9 targeting *bla*_CTX-M_ group 1 target II | This study |
| pRC319-G9 | Neo^R^/Km^R^ | Phagemid carrying CRISPR-Cas9 targeting *bla*_CTX-M_ group 9 | This study |
| pRC319-P | Neo^R^/Km^R^ | Phagemid carrying CRISPR-Cas9 targeting *bla*_CTX-M_ promoter | This study |
| pCCI | Cm^R^ | Cm^R^ vector | [36] |
| HP17_KO7 (Plasmid #120346) | Km^R^ | Helper plasmid | [35] |
| HP17_CO7 | Cm^R^ | Modified helper phage | This study |
