# Supplementary material for: Non-replicative phage particles delivering CRISPR-Cas9 to target major blaCTX-M variants
Source: PLoS One. 2024 May 16;19(5):e0303555. doi: 10.1371/journal.pone.0303555 (PMC11098365; doi:10.1371/journal.pone.0303555)
Supplement: S2 Table — (DOCX) [file pone.0303555.s005.docx]

**S2 Table. Primers used in this study.**

| **Primer name** | **Sequence (5'–3')** | **Feature** | **Annealing temperature (ºC)** | **Amplicon size (bp)** |
| --- | --- | --- | --- | --- |
| ISEcp1_Prom_F | GATTGAAAGGTGGTTG | Detection and cloning of *bla*_CTX-M_ group 1 and 9 | 60 | 1,100 |
| ISEcp1_Prom_R | GCTTTTACAAACCGTC | Detection and cloning of *bla*_CTX-M_ group 1 | 60 | 1,100 |
| ISEcp1_Prom_14_R | CCAGTTACAGCCCTTC | Cloning of *bla*_CTX-M_ group 9 | 60 | 1,100 |
| Spc_PspXI_F | ACTCGAGCGAACGCCCTAGGTCTAGG | Detection and cloning of CRISPR array targeting *bla*_CTX-M_ group 1, 9, and promoter | 58 | 341 and 421 |
| Spc_XmaI_R | CTGATACCCGGGACGCACTGACCGAATTC | Detection and cloning of CRISPR array targeting *bla*_CTX-M_ group 1, 9, and promoter | 58 | 421 |
| pCRISPR_G1_DT_R | CCGTGATACCACTTCACCTCGG | Detection of spacer targeting *bla*_CTX-M_ group 1 | 56 | 341 |
| pCRISPR_G9_DT_R | ACCGTTGCAGTACAGCGAC | Detection of spacer targeting *bla*_CTX-M_ group 9 | 56 | 341 |
| pCRISPR_promo_DT_R | CCCTTTCAATCATTTTTGATAAATCATTGATG | Detection of spacer targeting *bla*_CTX-M_ promoter | 56 | 341 |
| HP17_KO7-F | CCTGACTGGTATAATGAGCC | Detection of helper phage vector | 52 | 842 |
| HP17_KO7-R | GCGGAGTGAGAATAGAAAGG | Detection of helper phage vector | 52 | 842 |
| *Pvu*I_CmR_F | CGATCACGATCGTTACGCCCCGCCCTGC | Cloning of Cm^R^ gene into helper phage vector | 58 | 774 |
| *Dra*III_CmR_R | CGCACACGTTGTGTGATCGGCACGTAAGAGGTTCC | Cloning of Cm^R^ gene into helper phage vector | 58 | 774 |
