# Supplementary material for: Non-replicative phage particles delivering CRISPR-Cas9 to target major blaCTX-M variants
Source: PLoS One. 2024 May 16;19(5):e0303555. doi: 10.1371/journal.pone.0303555 (PMC11098365; doi:10.1371/journal.pone.0303555)
Supplement: S3 Table — (DOCX) [file pone.0303555.s006.docx]

**S3 Table.** **Target sequences of the predominant *bla*_CTX-M_ group 1, group 9, and promoter for construction of spacer.**

| **Target** | **Spacer and PAM sequence (5'-3')** | **Protospacer adjacent motif (PAM)­­** | **Designed oligonucleotide for CRISPR array (5'-3')** |
| --- | --- | --- | --- |
| *bla*_CTX-M_ group 1_I | GCGCGGCCGCGCTACAGTACAGCGATAACGTGG | TGG | Oligo 1- AAACGCGCGGCCGCGCTACAGTACAGCGATAACGG  Oligo 2- AAAACCGTTATCGCTGTACTGTAGCGCGGCCGCGC |
| *bla*_CTX-M_ group 1_II | CGCCATTGCCCGAGGTGAAGTGGTATCACGCGG | CGG | Oligo 1- AAACCGCCATTGCCCGAGGTGAAGTGGTATCACGG  Oligo 2- AAAACCGTGATACCACTTCACCTCGGGCAATGGCG |
| *bla*_CTX-M_ group 9 | TCATGGCGGTATTGTCGCTGTACTGCAACGCGG | CGG | Oligo 1- AAACTCATGGCGGTATTGTCGCTGTACTGCAACGG  Oligo 2- AAAACCGTTGCAGTACAGCGACAATACCGCCATGA |
| *bla*_CTX-M_ promoter | ATCAATGATTTATCAAAAATGATTGAAAGGTGG | TGG | Oligo 1- AAACATCAATGATTTATCAAAAATGATTGAAAGGG  Oligo 2- AAAACCCTTTCAATCATTTTTGATAAATCATTGA |
